# Space State Representation Product Evaluation in Satellite Position and Receiver Position Domain

**DOI:** 10.3390/s20133791

**Published:** 2020-07-06

**Authors:** Renata Pelc-Mieczkowska, Dariusz Tomaszewski

**Affiliations:** Faculty of Geoengineering, University of Warmia and Mazury in Olsztyn, Chair of Geoinformation and Cartography, 10-719 Olsztyn, Poland

**Keywords:** SSR stream, IGS, satellite clock corrections

## Abstract

In Global Navigation Satellite Systems (GNSS) positioning, important terms in error budget are satellite orbits and satellite clocks correction errors. International services are developing and providing models and correction to minimize the influence of these errors both in post-processing and real-time applications. The International GNSS Service (IGS) Real-Time Service (RTS) provides real-time orbits and clock corrections for the broadcast ephemeris. Real-time products provided by IGS are generated by different analysis centres using different algorithms. In this paper, four RTS products—IGC01, CLK01, CLK50, and CLK90—were evaluated and analysed. To evaluate State Space Representation (SSR) products’ GPS satellites, the analyses were made in three variants. In the first approach, geocentric real-time Satellite Vehicle (SV) coordinates and clock corrections were calculated. The obtained results were compared with the final IGS, ESA, GFZ, and GRG ephemerides. The second approach was to use the corrected satellite positions and clock corrections to determine the Precise Point Position (PPP) of the receiver. In the third analysis, the impact of SSR corrections on receiver Single Point Position (SPP) was evaluated. The first part of the research showed that accuracy of the satellite position is better than 10 cm (average 3 to 5 cm), while in the case of clock corrections, mean residuals range from 2 cm to 17 cm. It should be noted that the errors of the satellites positions obtained from one stream differ depending on the reference data used. This shows the need for an evaluation of correction streams in the domain of the receiver position. In the case of PPP in a kinematic mode, the tests allowed to determine the impact that the use of different streams has on the final positioning results. These studies showed differences between specific streams, which could not be seen in the first study. The best results (3D RMS at 0.13 m level) were obtained for the CLK90 stream, while for IGC01, the results were three times worse. The SPP tests clearly indicate that regardless of the selected SSR stream, one can see a significant improvement in positioning accuracy as compared to positioning results using only broadcast ephemeris.

## 1. Introduction

Positioning based on Global Navigation Satellite Systems (GNSS) is now a natural element of our everyday life. The choice of positioning method is determined on the one hand by expectations of precision, while limited by measurement conditions, equipment, and resources on the other hand. Many of the GNSS measurement budget errors can be eliminated or mitigated using differential methods. The limitation in using differential methods is the need to provide access to a relatively close (maximum of several dozen kilometres’ distance) reference station or a network of such stations. To overcome this limitation, Precise Point Positioning (PPP) methods in post-processing mode as well as in real-time are being developed [1]. To step forward from real-time SPP (code-only solution) to real-time PPP (code + phase solution) with position precision of the decimetre or even centimetre-level, it is necessary to have access to several, high-quality external corrections. These external corrections are created to mitigate or even eliminate most kind of errors and take full advantage of the high precision phase and code observations [2,3,4]. 

In addition to the ionospheric and tropospheric delay, multipath, receiver noise, and resolution errors, one of the significant items in GPS error budgets are errors related to the space segment in general, satellite clock errors, and ephemerides errors. The broadcast GPS ephemeris has 1-m level satellite positioning accuracy and broadcast clock parameters provide accuracy at the level of 5 ns [5]. Therefore, to obtain more precise real-time position, it is necessary to provide corrections to the GPS broadcast ephemeris and satellite clocks corrections. 

The IGS service began to work on enabling real-time SSR in 2001 by establishing Real-Time Working Group (RTWG) [6,7]. The RTS clock and orbit corrections were officially available from the year 2013. From that point on, GPS and GLONASS systems users have been able to use IGS streams to perform real-time PPP measurements for a verity of applications. In mid-2011, the IGS initiated the Multi-GNSS Experiment (MGEX), which was aimed at generating products for all GNSS constellations [8]. Nowadays, for Galileo, BeiDou, and QZSS systems, IGS Final and Rapid orbits and clock are only usable for post-processing applications [9]. 

Currently, there are several main broadcasters that enable RTS corrections for broadcast GPS ephemeris and clock data, which are International GNSS Service (IGS) and analysis centres (ACs): Bundesamt für Kartographie und Geodäsie (BKG), WUHAN University (WHU), European Space Agency (ESA/ESOC), Centre National d’Études Spatiales (CNES), Deutsches Zentrum Fur Luft – und Raumfahrt (DLR), GMV Aerospace and Defense, and Natural Resources Canada (NRCan). A list of the streams and their brief descriptions is presented in Table 1 [10]. System users can take advantage of individual solutions or use streams created by combining other SSR solutions like IGS. These combinations are made based on single solutions sent from centres, calculating the corrections around the world. The IGS real-time services provide four such SSR streams [10,11].

Based on Table 1, one can notice that single SSR solutions are sent by the centres in pairs, for the centre of satellite mass (CoM) and antenna phase centre (APC). Most of the SSR corrections are computed with the use of RETINA software developed by ESA [12]. 

Theoretically, solutions sent by all institutions should give similar results. Practically positioning performances could be different when different RTS products are applied [2]. To assist the user in making an informed decision on the selection of the RTS stream from among those available, evaluations of these products are conducted. These evaluations focus on two main aspects: availability and accuracy. 

In the area of availability, two requirements for the RTS products should be distinguished. The first one is that the RTS stream can be received at the given epoch. According to Hadaś and Bosy [13], the availability of IGS GPS correction streams was over 95% and most data gaps were shorter than 1 h. The availabilities of IGS RTS streams as well as those provided by individual ACs (based on nine products IGS01, IGS03, CLK01, CLK15, CLK22, CLK52, CLK70, CLK81, CLK90 investigation) have been comprehensively analyzed by Zhang et al. [11]. According to their results, daily epoch availabilities of all RTS products are also high and reach 90%. The second requirement is that latency is smaller than a specific threshold. The size of this threshold is closely related to the rate of RTS product accuracy degradation over time. For the GPS system, it oscillates around 1 to 2 cm/min in case of radial, along and cross-track component and reaches 10 cm/min in case of clock correction. Hence, an additional 5-centimetre error is expected when using orbit corrections with 3 min latency and clock correction with 1 min latency. Reliable prediction of the GPS orbit correction is possible up to 8 min using polynomial fitting of radial, along and cross-track SSR components and clock corrections [13] or by predicting geocentric orbit corrections [14]. The average latency obtained from one day of observation varies depending on the product tested from a few seconds (CLK01, CLK15, CLK22, CLK81, and CLK90) to almost half a minute (IGS01 and IGS03) [11]. The nominal accuracy of RTS products is mainly assessed using Final orbit and clock products as reference. Previous research has shown that the RMSE (Root Mean Square Error) of radial, along-track, and cross-track components varies slightly depending on the product. Hadaś and Bosy demonstrated that the radial, along and cross-track RMS of the IGS RTS products does not exceed 2, 4, and 3 cm, respectively, for GPS satellites (IGC01) and 3, 10, and 8 cm for GLONASS satellites (IGC03) [11]. Wang et al. analyzed streams generated by various ACs and according to that research, CLK51 performed the best in the RMS comparison (2.09, 3.29, and 2.74 cm in the radial, along, and cross-track direction) while IGS products were the worst (5.79, 8.04, and 7.02 cm, again in those three directions). In case of GLONASS satellites, RTS products were almost two times worse [15]. Accuracy of GPS clock products and GLONASS clock products is about 8 cm and 13 cm, respectively [11,13,16]. Kaźmierski et al. and Wang et al. have also analysed Galileo and BeiDou RT orbit and clock corrections provided by CNES in addition to GPS and Galileo SSR RTS. They both confirmed the highest accuracy of GPS orbits. The accuracy for BeiDou real time orbits is the worst within in 8, 15, and 19 cm in radial, along, and cross-track direction [15,16]. However, the results of the above-mentioned work differ in the case of Galileo. Wang et al. showed that the accuracy of these orbits is slightly worse than GPS but much better than BeiDou and is 3, 5, and 4 cm respectively, while Kaźmierski et al. obtained results over twice worse (6, 12 and 20 cm). This difference is probably due to the use of different final products as a reference, which will be discussed in this paper.

It should be strongly emphasized that IGS final orbits and clocks were used as reference data in all the works mentioned (except Galileo and DBS orbits, where the final CODE MGEX solution [16] or final precise GMB product released by GFZ [15] is employed), while each ACs provides their own final orbits and clocks. Similar to the case of RTS streams, these products are calculated using different software based on different algorithms, due to which provided data also differ from each other. The authors of the previously mentioned papers, while choosing only one source of reference data, did not take these differences into consideration. Considering only one source of reference data may result in faulty results during comparative analysis of streams. Therefore, appropriate evaluation of SSR products requires the use of reference data from different ACs. 

The aim of this work is to thoroughly evaluate representative SSR products both in the satellite position domain and in the receiver position domain. When analyzing the accuracy of positioning, the benefits of using SSR in both PPP positioning and the SPP method was assessed.

In the first part of this study, the authors performed an evaluation of GPS RTS streams generated by various ACs taking as reference data IGS final products as well as those generated by individual ACs (peer-to-peer analysis). 

Both the orbitals and satellite clock corrections have a significant effect on positioning result. From the system user’s point of view, the final error in determining the receivers position is the most important. Hence the other, indirect method of RTS product evaluation is based on testing accuracy in the domain of the receiver position. Wang et al., based on a 20 h experiment with 10 IGS stations, stated that simulated, 3D position error does not exceed 10 cm, and is the best for CLK93 (6.9 cm 3D RMS) [15], while Kaźmierski et al. achieved 6 cm 3D accuracy using the multi-GNSS mode [16]. The mentioned tests were performed on relatively short samples of data. According to the authors, correct evaluation of the method requires multiple position determination with repeated changes in the satellites constellation and measurement conditions. This task has been performed in the second part of the presented study where PPP positioning was conducted during the 33-day experiment, giving almost 3 million positioning results for each station.

The last analysis was done with the use of SPP positioning because this aspect of positioning is usually overlooked in terms of SSR corrections. It should be noted that most currently used receivers use only absolute positioning which could be improved by accessing SSR corrections.

For the presented research, the authors chosen four Real-Time Service products: IGC01, CLK01, CLK50, and CLK90 to be evaluated and analysed. Each of these streams were generated by different ACs (IGS, BKG, ESA/ESOC, and CNES). As a reference data for RTS orbits, four final products generated by mentioned ACs were used. Different ACs use different sets of permanent reference stations and different software (RETINA, RTNet, PPP-WIZARD), which causes differences in the generated products. The RETINA software computes precise orbits and clocks with the use of batch data pre-processing [17]. RTNet processes zero-differenced observations and the satellite and receiver clock corrections are estimated at every epoch independently with the use of square-root filter [18]. The PPP-WIZARD processing was based on a Kalman filter with undifferenced ambiguity resolution [19]. The latency of CLK90 is about 8 s and of IGC01 is above 24 s according to Rülke [20]. In addition, the IGC01 stream is single epoch combination of solutions (CLK10, CLK16, CLK20, CLK22, CLK53, CLK70, CLK80), while the other streams are individual solutions. Note that none of the other selected streams are part of the IGC01 combination. The data contained in tested streams concern satellite centre of mass. The update rate for selected streams is 10 s for IGC01 and 5 s for CLK01, CLK50, and CLK90. After analysing the accuracy of individual SSR streams, an analysis of the impact of using considered streams in the domain of the receiver position PPP was made.

## 2. Real-Time Service (RTS) Satellite Space State Representation

Space State Representation aims to minimize the errors of the GNSS space segment by providing satellite position and clock corrections with precise ephemeris. Accurate satellite coordinates X_satellite_ (vector of coordinates) in ECEF frame are calculated by subtracting satellite position corrections δX computed from SSR data and broadcast ephemeris X_brodcast_. GPS real-time clock corrections have a form of a third-degree polynomial. Both information is transmitted as an RTCM stream (Message types 1058, 1060) [20]. Streamed parameters are used to calculate value δC and δX, which are applied to broadcast satellite orbit and clock. The value of corrections is calculated as follows [13,21,22,23]:

(1) $$\begin{array}{c} X_{\mathrm{satellite}\;}={\;X}_{\mathrm{broadcast}}-\;\delta X\\ t_{\mathrm{satellite}\;}={\;t}_{\mathrm{broadcast}}-\frac{\delta C}{c} \end{array}$$

where $X_{\mathrm{broadcast}}$—vector of satellite coordinates computed from broadcast data message, $t_{\mathrm{broadcast}}$—satellite time computed according to corresponding GNSS ICD from broadcast clock parameters, identified by IOD/IODE of corresponding SSR Correction message, $X_{\mathrm{satellite}\;}$—vector of satellite coordinates corrected by SSR Correction message, $t_{\mathrm{satellite}}$—satellite time corrected by SSR Clock Correction message, δC—clock correction obtained from SSR Clock Correction message, δX—satellite position correction obtained from SSR Correction message, c—speed of light (c = 299 792 458 m/s).

Value of satellite position correction δX is calculated according to the algorithm:

(2) δX=EδO

where δO—orbit corrections vector, E—matrix containing unit radial ($e_{r}$), along ($e_{a}$) and cross ($e_{c}$) vectors.

(3) $$E=\left[\begin{array}{ccc} e_{r}^{X} & e_{r}^{Y} & e_{r}^{Z}\\ e_{a}^{X} & e_{a}^{Y} & e_{a}^{Z}\\ e_{c}^{X} & e_{c}^{Y} & e_{c}^{Z} \end{array}\right]$$

Values of unit vectors are calculated from broadcast data message:

(4) $$\begin{array}{c} e_{a}=\frac{\dot{r}}{\left|\dot{r}\right|}\\ e_{c}=\frac{r\times \dot{r}}{\left|r\times \dot{r}\right|}\\ e_{r}={\;e}_{a}\times {\;e}_{c} \end{array}$$

where $r={\;X}_{\mathrm{broadcast}}$

$\dot{r}={\dot{X}}_{\mathrm{broadcast}}$- vector of satellite velocities computed from broadcast data message.

The value of orbit correction vector δO consists of correction terms (${\left[\begin{array}{ccc} {\delta O}_{r} & {\delta O}_{a} & {\delta O}_{c} \end{array}\right]}^{T}$) and its rates (${\left[\begin{array}{ccc} {\delta \dot{O}}_{r} & {\delta \dot{O}}_{a} & {\delta \dot{O}}_{c} \end{array}\right]}^{T}$) are obtained from the SSR message:

(5) $$\delta O={\;\delta O}_{0}+\delta \dot{O}=\left[\begin{array}{c} {\delta O}_{r}\\ {\delta O}_{a}\\ {\delta O}_{c} \end{array}\right]+\left[\begin{array}{c} {\delta \dot{O}}_{r}\\ {\delta \dot{O}}_{a}\\ {\delta \dot{O}}_{c} \end{array}\right]\left(t-t_{0}\right)$$

Value of SSR clock correction δC is calculated according to the formula:

(6) $$\delta C={\;C}_{0}+{\;C}_{1}\left(t-t_{0}\right)+C_{2}{\left(t-t_{0}\right)}^{2}$$

where:
t—GPS measurement time
$t_{0}$—reference time obtained from SSR Clock Correction message
$C_{0},C_{1},C_{2}$—polynomial coefficients from SSR Clock Correction message

Reference time $t_{0}$ is calculated based on correction time ($t_{\mathrm{corr}}$) and half of the SSR update interval ($t_{\mathrm{SSRint}}$):

(7) $$t_{0}=t_{\mathrm{corr}}+\frac{t_{\mathrm{SSRint}}}{2}.$$

Additionally, for GPS observations, the relativistic correction (${\left(\Delta t\right)}_{r}$) must be applied to compute satellite time from broadcast ephemeris ($t_{\mathrm{broadcast}}$):

(8) $${\Delta t}_{r}=\frac{2\;r\;\dot{r}}{c^{2}}$$

where $r,\dot{r}$ are satellite position and velocity vectors computed from the broadcast ephemeris.

## 3. Evaluation of SSR Correction Streams

In the study, positions and clock corrections were determined for each of the available GPS satellites. Thirty-one days of logged correction streams were used for the experiment. During the experiment, approx. 11 million position determinations and satellite clock corrections for selected streams were made. SSR orbit and clock corrections computations were made using own software. PyGNSS software enables to use six data streams simultaneously. Each set of SSR corrections were calculated in real-time using four selected streams: IGC01, CLK01, CLK50, and CLK90. The obtained results were recalculated and refer to the International Terrestrial Reference Frame 2014 (ITRF2014). Calculated values of the SV coordinates and SV clocks corrections were compared with four selected final ephemeris and clock products:

- IGS generated by International GNSS Service
- ESA generated by European Space Agency
- GFZ generated by German Research Centre for Geosciences
- GRG generated by Centre National d’Études Spatiales

**Figure 1:**
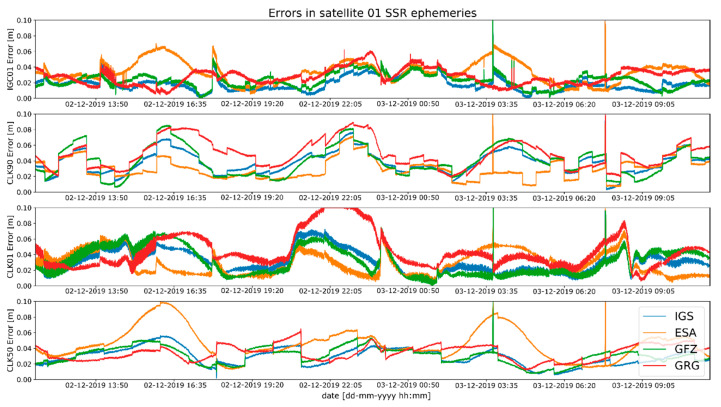
Satellite Vehicle Number (SVN) 01 SSR positioning errors for the tested Radio Technical Commission for Maritime Services (RTCM) streams.

**Figure 2:**
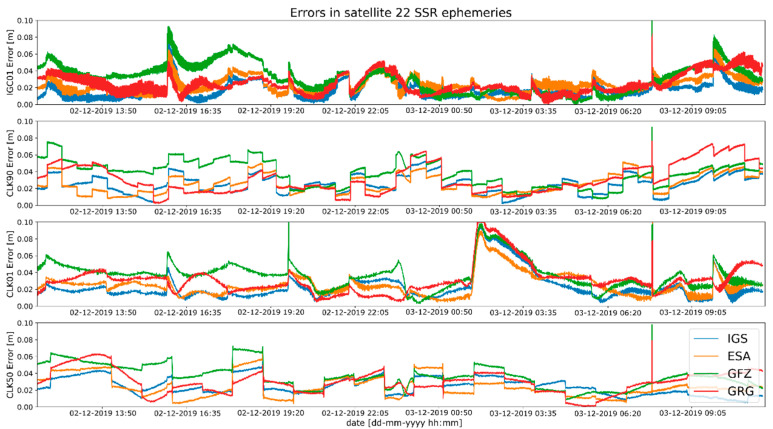
SNV 22 SSR positioning errors for the tested RTCM streams.

Analysis of the positions and clocks accuracy was carried out based on various reference data. Such analysis allowed for reliable assessment of individual streams’ accuracy. Otherwise, it is hard to avoid a situation where the SSR stream and final ephemeris are calculated by the same analysis centre. Additionally, the adopted strategy allows to determine whether the data generated by the same centres are most compatible with each other. Geometric distances between the SSR corrected satellite coordinates (X_satellite_) and SP3 final coordinates at the time of measurement (t) were assumed as a residual of satellite coordinates. Sample SV position residuals calculated for satellite SVN 01 and SVN 22 are presented in Figure 1 and Figure 2.

A cursory analysis of Figure 1 and Figure 2 may suggest that results from individual SSR streams seem to be similar. However, the characteristics of the waveform, shape, and variability are different for residuals for each individual stream. At the same time, the order of magnitude of the residuals obtained is similar for each SSR stream. The difference in these characteristics is due to the different ways of obtaining the solution used in software for each AC (single epoch, combination or Kalman filter). However, it should be noted that for the same SSR stream, the residuals are different depending on the selected reference data. For example, for the SVN 01 satellite, the CLK 90 stream has the lowest residual values relative to the orbit calculated by ESA. While at the same time for the CLK 50 stream, the residual values were the highest for the ESA orbit and the lowest for the IGS orbit. A similar dependence can be observed for all streams with different satellites.

**Figure 3:**
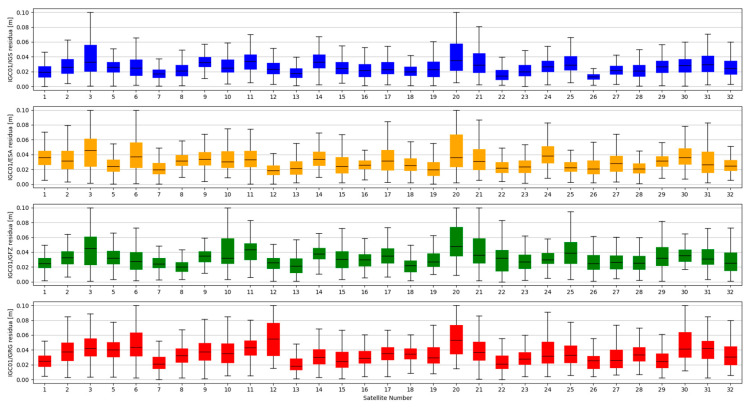
Statistical summary of State Space Representation (SSR) satellite position residuals for the IGC01 SSR stream.

**Figure 4:**
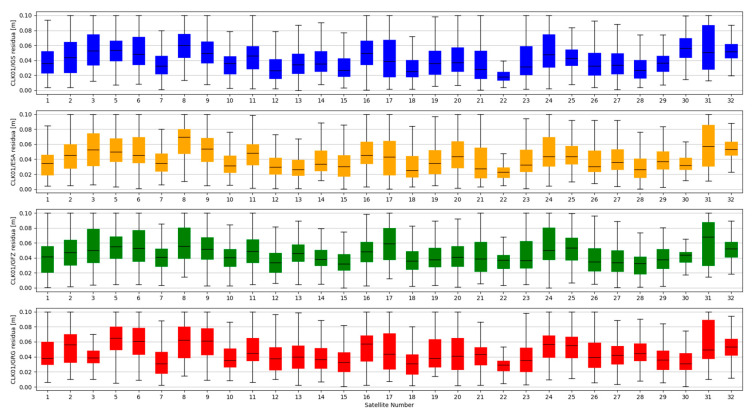
Statistical summary of SSR satellite position residuals for the CLK01 SSR stream.

**Figure 5:**
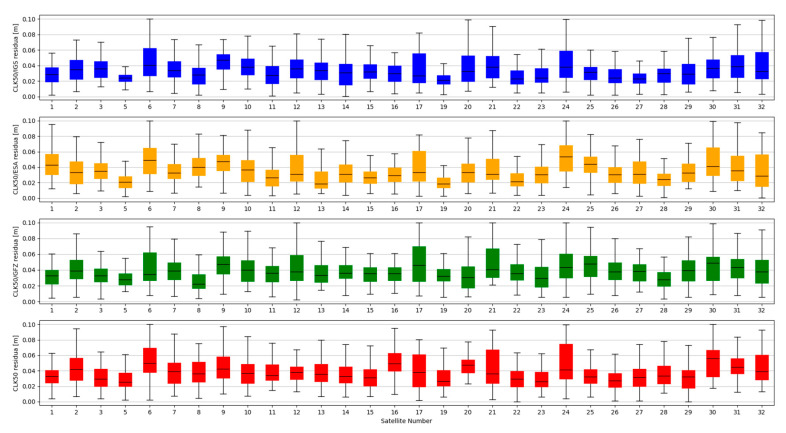
Statistical summary of SSR satellite position residuals for the CLK50 SSR stream.

**Figure 6:**
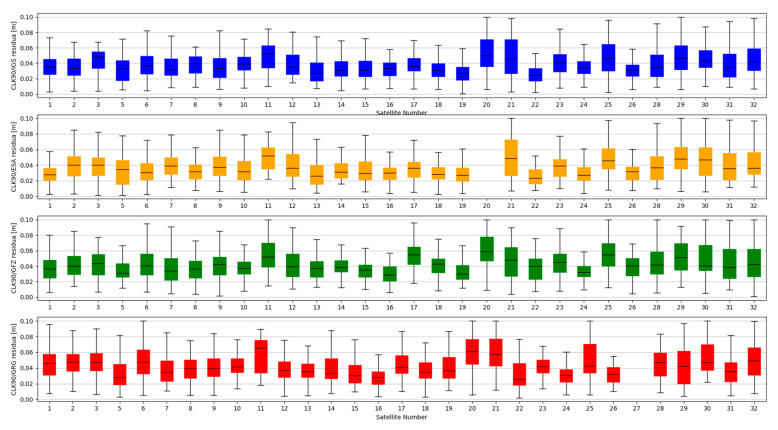
Statistical summary of SSR satellite position residuals for the CLK90 SSR stream.

Studies of satellites position residuals were carried out in 1-s interval for all available satellites in space. Basic statistical data was calculated for all obtained results (Table 2). The distribution of the received satellite position residuals is shown in Figure 3, Figure 4, Figure 5 and Figure 6. Each figure shows the satellite position residual values of different SSR corrections with respect to four selected reference final ephemerides (IGS—Blue, ESA—orange, GFZ—green, GRG—red).

The mean value of satellite position error, calculated based on broadcast ephemeris, is about 1 m [24]. The results presented above show that the use of SSR streams significantly reduces the value of the SV position error. After State Space Representation corrections were applied, the positions residuals relative to the final ephemeris were reduced to a few centimetres. Based on the analysis of Figure 3, Figure 4, Figure 5 and Figure 6, it can be concluded that the residual values are similar, irrespective of the reference ephemerides used. However, numeric values contained in Table 2 show some dependence. In case of IGS ephemerides, the smallest mean satellite position error value was recorded for the IGC01 stream (0.027 m). For ESA final ephemerides, the smallest value was 0.032 m for CLK50 stream (both calculated by ESA). The lowest average residual value for GFZ (0.033m) is again the IGC01 stream. In case of GRG, the smallest value was 0.036 for the CLK90 stream (both calculated by CNES). For all reference ephemerides, the CLK01 stream proved to have the smallest satellite position accuracy. As assumed, the satellite positions determined from the final products most often match the results from real-time streams generated by the same centre. However, the IGC01 stream had quite low values for all reference data. It should also be noted that the differences between the results obtained are small and will not have a significant impact on the final positioning results.

**Figure 7:**
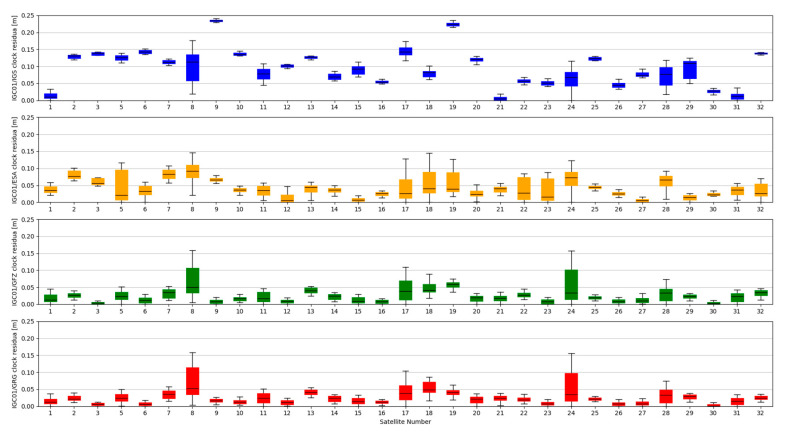
Statistical summary of SSR satellite clock residuals for the IGC01 SSR stream.

**Figure 8:**
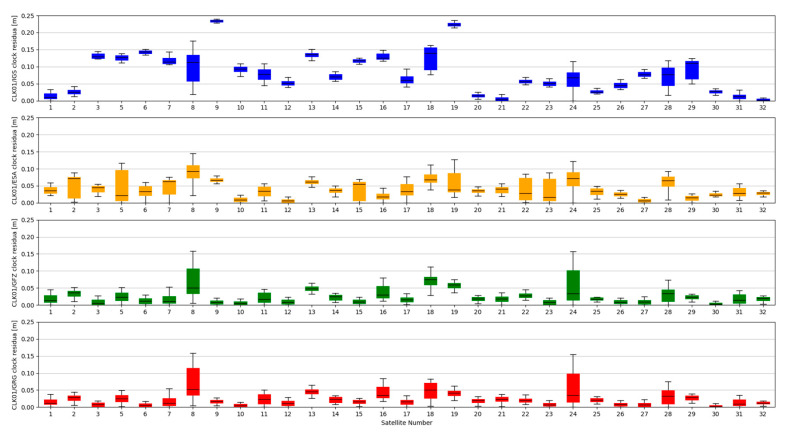
Statistical summary of SSR satellite clock residuals for the CLK01 SSR stream.

**Figure 9:**
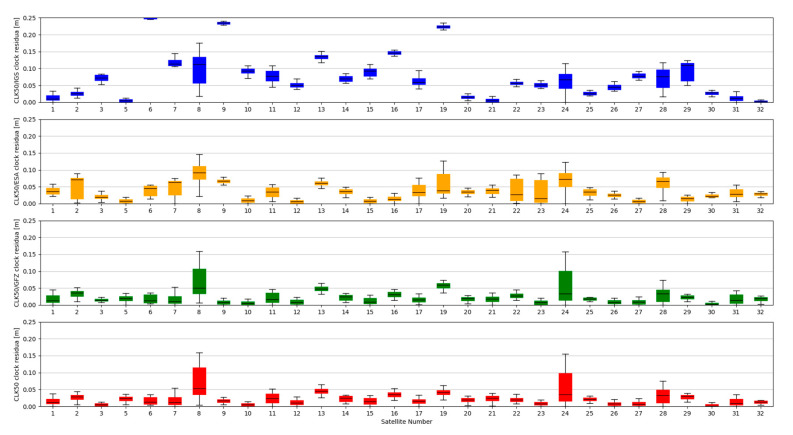
Statistical summary of SSR satellite clock residuals for the CLK50 SSR stream.

**Figure 10:**
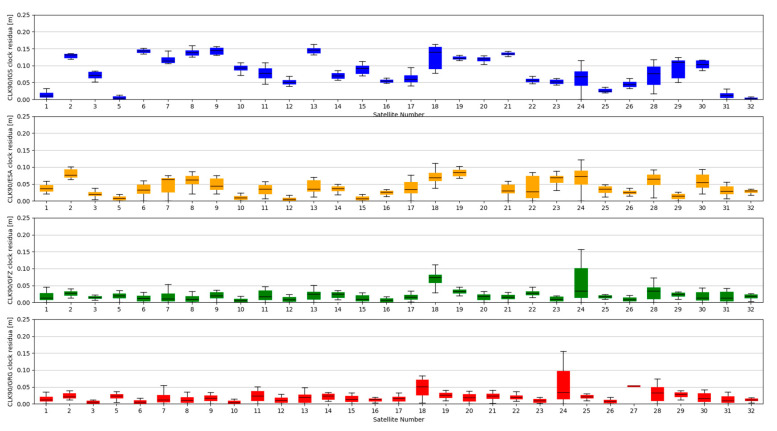
Statistical summary of SSR satellite clock residuals for the CLK90 SSR stream.

The accuracy of satellite clock correction is very important in the case of precise positioning. The second study carried out an analogous analysis of the satellite clock corrections using the same reference data. To evaluate real-time clock corrections, in each epoch, a reference satellite was selected. Subsequently, individual single clock differences between the reference satellite and other satellites were determined. These values were calculated for both final precise clocks and for the values obtained from individual RTCM streams. Clock residuals are defined as differences in the obtained single clock difference in respective epochs, between final products and SSR corrections stream. Figure 7, Figure 8, Figure 9 and Figure 10 depicts the residuals distribution of the GPS real-time clock products with respect to the IGS, ESA, GFZ, and GRG final clock correction.

The analysis of the accuracy of the satellite clock corrections shows different dependencies than in the case of the satellite position. The IGC01 stream has the highest residual values for all reference values used. At the same time, the lowest residual values for ESA and GRG clocks have the same streams, as in the case of satellite position analyses, generated by the same Analysis Centre (CLK50 and CLK90). In case of GFZ, the CLK90 stream was recorded with the smallest residual values. Based on all collected data, it can be stated that the CLK90 and CLK50 streams have similar and the highest accuracy for all analysed cases (Table 3).

Research conducted in this part of the article does not allow to draw clear conclusions. For both analyses, depending on the reference data, the different streams appear to have the highest accuracy. Therefore, subsequent analyses were carried out to determine the positioning accuracy, in the receiver position domain.

## 4. Evaluation of SSR Corrections Streams in the Receiver Position Domain—PPP Solution

**Figure 11:**
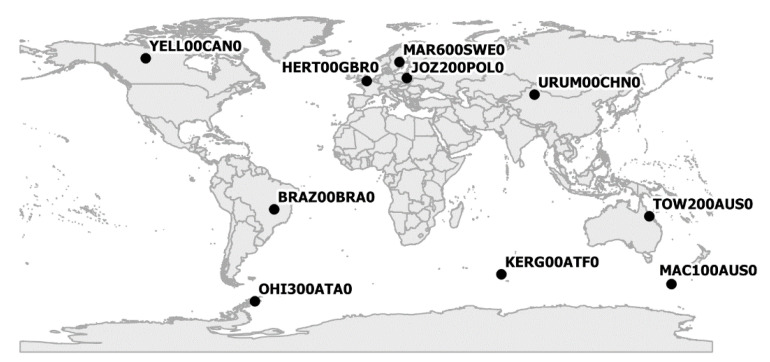
Localisation of test stations.

From the GPS system user point of view, one of the most important positioning aspects is the final accuracy. The analyses presented in Section 3 do not allow to select the SSR stream that will achieve the highest accuracy. Therefore, positioning analysis was performed to check the impact of different SSR corrections on the result from the PPP algorithm. For this purpose, PPP positions of 10 permanent IGS stations were determined in kinematic mode. Test stations were selected in such a way that they were distributed at different latitudes and longitudes (Figure 11). The placement of the stations ensured checking if the use of SSR corrections enabled the same positioning accuracy, regardless of the position of the receiver. Station positions were determined continuously over 33 days. For each station, raw observational data in the form of RTCM streams was used. Positioning was performed for four variants; in each of them, a SSR RTS generated by another AC was used (IGC01, CLK01, CLK50, CLK90). For all streams, the same ANTEX file and troposphere model, as well as processing parameters were used (Table 4).

**Figure 12:**
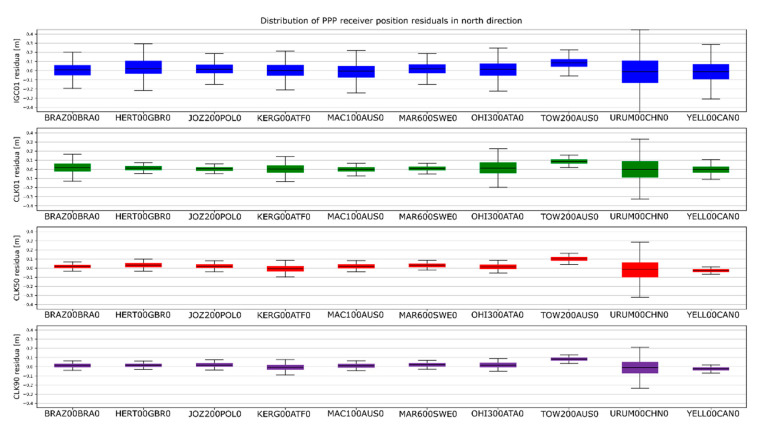
Distribution of PPP residuals in the north direction.

**Figure 13:**
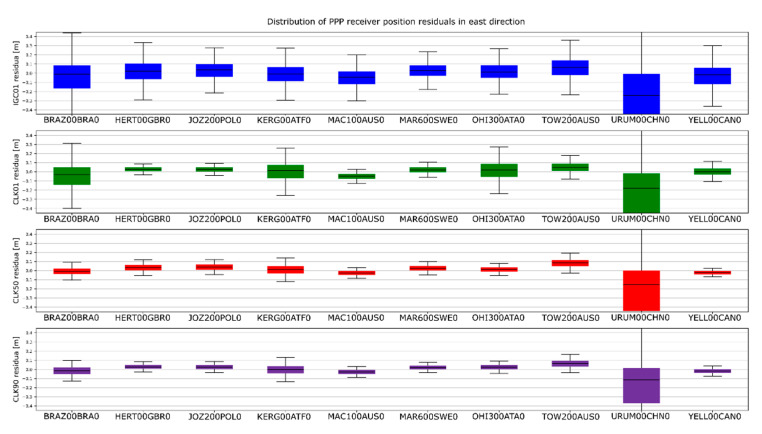
Distribution of PPP residuals in the east direction.

**Figure 14:**
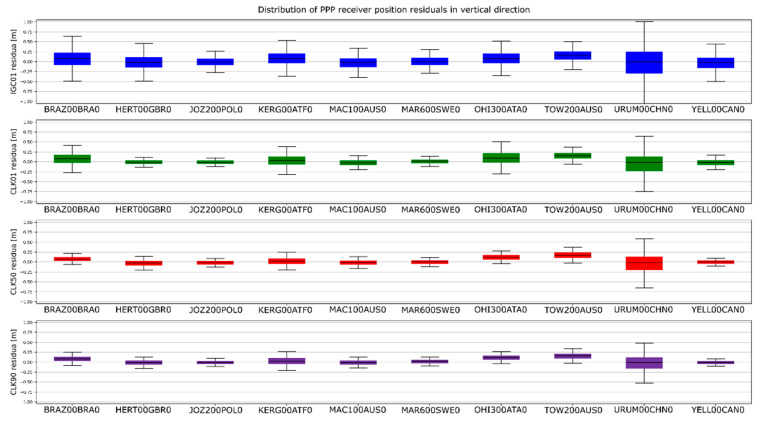
Distribution of PPP residuals in the vertical direction.

The determinations carried out were converted into ENV (East, North, Vertical) coordinates. The obtained results were subtracted from known coordinates of the IGS stations. Finally, four sets of position residua, for each station, were used in further analyses (Figure 12, Figure 13 and Figure 14).

Figure 12, Figure 13 and Figure 14 present the distribution of position residuals in north, east, and vertical directions for select IGS stations. The values obtained for the IGC01, CLK01, CLK50, and CLK90 streams are depicted in blue, green, red, and violet, respectively.

**Figure 15:**
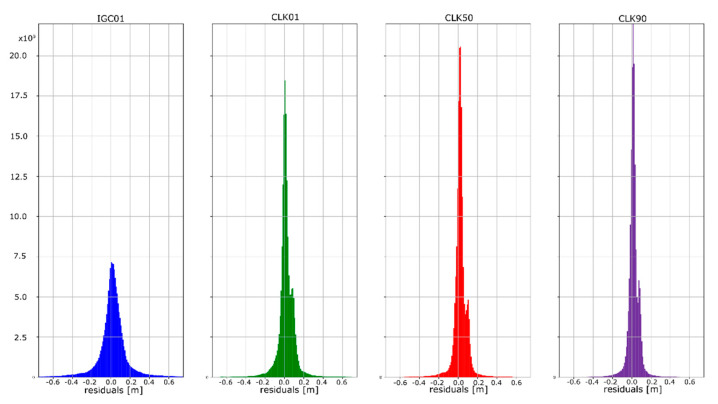
Histogram of positioning residuals in the north direction for the selected stations.

**Figure 16:**
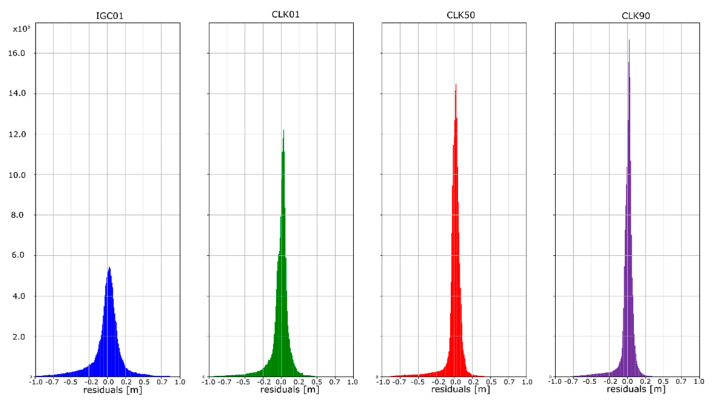
Histogram of positioning residuals in the east direction for the selected stations.

**Figure 17:**
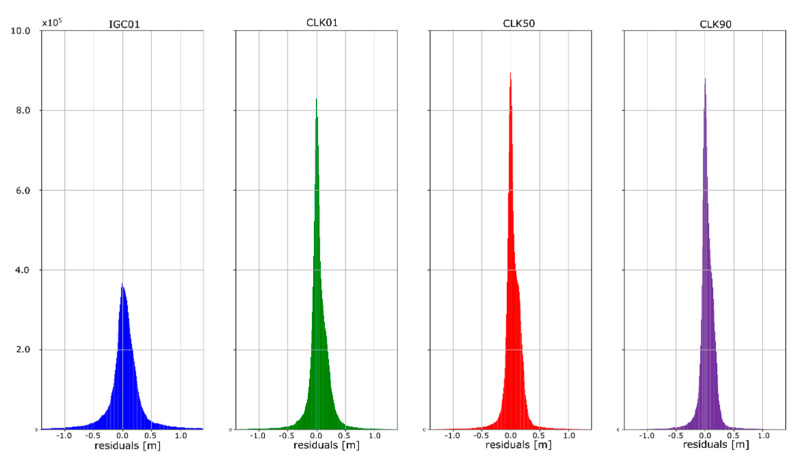
Histogram of positioning residuals in the vertical direction for the selected stations.

Unlike the analysis of the satellite coordinates and clocks, significant differences in the results can be observed in this study. For all streams, the largest positioning residuals were observed for the results from the URUM00CHN0 station, because there are many terrain obstacles around and the station also faced technical problems with the Javad Triumph receiver while the experiment was conducted. If we look at the residuals of positions determined from weekly observations by IGS, in the case of the URUM stations, they are on average two times larger than in the case of other stations. In the case of stations stream CLK90 and CLK50, the residuals’ boundary is much tighter than in the case of the other streams. This is especially noticeable for stations YELL00CAN0, URUM00CHN0, and BRAZ00BRA0. In the case of the IGC01 stream, the largest position residuals can be observed in all directions. For all stations tested, the positioning residuals’ distribution for the IGC01 stream is more than twice worse than for the other streams. Higher positioning residuals can also be observed in case of stations BRAZ00BRA0, KERG00ATF0, and OHI300ATA0 when the CLK01 stream was used. Such values were not found in the case of streams CLK50 and CLK90. Figure 15, Figure 16 and Figure 17 present a histogram of all the obtained residual values for the selected streams.

The mean absolute value of horizontal position residuals for the IGC01 value is 0.123 m dN and 0.164 m dE. For the CLK01 and CLK50, these values were two times smaller and amounted to 0.063 m and 0.052 m dN, and 0.094 m and 0.070 m dE. For the CLK90 stream, the values of average horizontal component residuals are even lower and equal 0.044 m dN and 0.067 m dE (Table 5). The IGC01 stream is also characterized by the largest standard deviation (0.222 m) when it is 0.168 m (CLK01. CLK50) and 0.0120 m (CLK90) in the other tested variants. Differences between statistical values obtained for 2, 3, and 4 variants oscillate in centimetre values. However, the CLK 90 stream showed the highest horizontal accuracy. In the case of the vertical component, comparing the obtained values gives similar conclusions as the horizontal position analyses. In the case of the IGC01 stream, mean absolute value of vertical component residuals is 0.320 m with 0.359 m standard deviation. While for CLK01 and CLK50, the mean absolute vertical value of 0.133 and 0.114 m was obtained with a standard deviation not exceeding 0.224 m. In the case of the vertical component, the best statistics were obtained using the CLK90. In this case, the mean value was 0.104 m with a standard deviation of 0.190 m (Figure 15, Figure 16 and Figure 17). An analysis of the PPP residuals distribution confirms previous considerations. Test results presented in the second study indicate that the highest accuracy was achieved with the CLK90 stream.

## 5. Evaluation of SSR Corrections Streams in the Receiver Position Domain—SPP Solution

**Figure 18:**
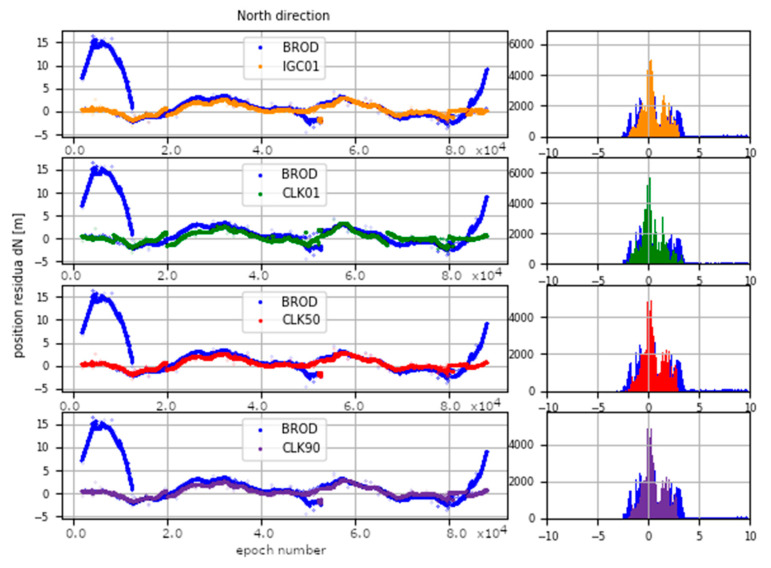
SPP residuals in the north direction.

**Figure 19:**
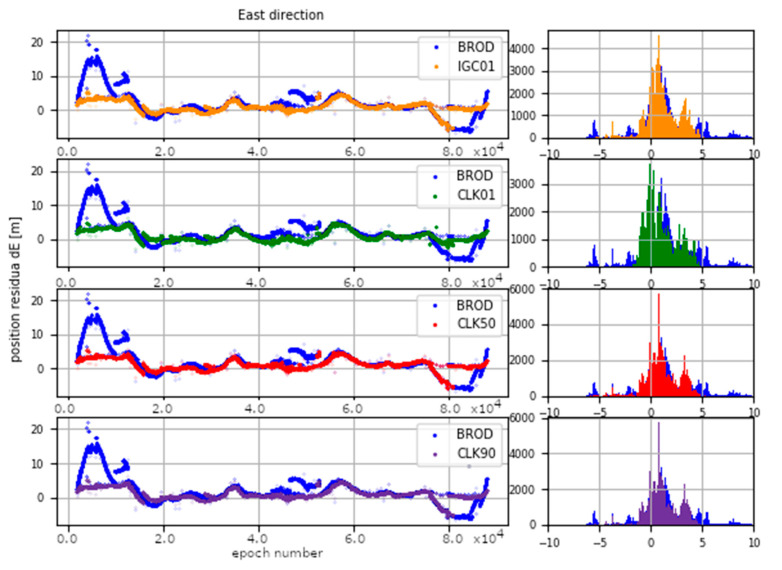
SPP residuals in the east direction.

**Figure 20:**
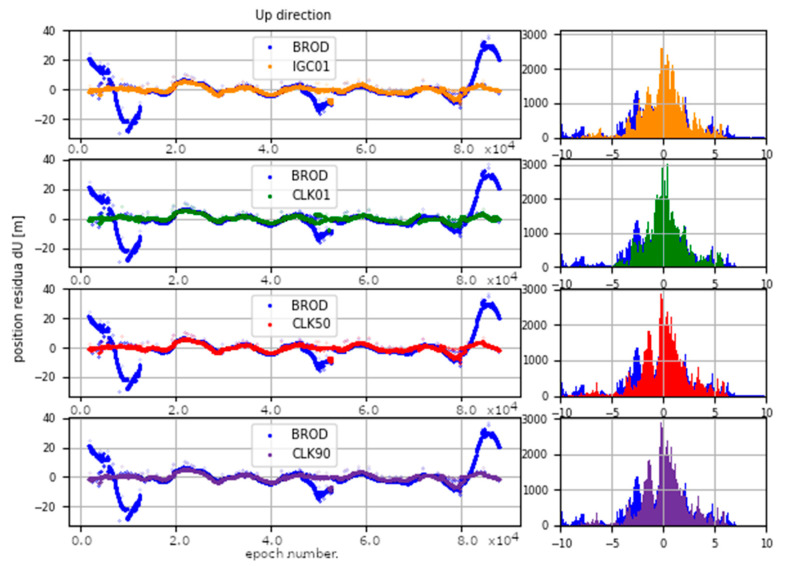
SPP residuals in the vertical direction.

The last analysis was a study performed to check the impact of SSR corrections on the result of SPP positioning. For this purpose. SPP positions were determined from a 24-h measurement session using the HERT00GBR0 station. with 1 s measurement interval. Positioning was performed for 5 variants. In the first variant. considered as reference. positioning was performed using the broadcast ephemeris. In the other four variants. the evaluation of positioning results using previously studied RTCM streams (IGC01. CLK01. CLK50. CLK90) was assumed. During the calculations. the cut-off angle was set to 0° and no SNR/C-N0 filtration was used. The UNB3m troposphere model was used to calculate wet and dry components. No ionospheric correction was determined. As in the PPP study. the determinations carried out were converted into ENV coordinates. The obtained results were subtracted from known coordinates of the IGS station. Finally. five sets of position residuals were received and further analysed (Figure 18, Figure 19 and Figure 20).

**Figure 21:**
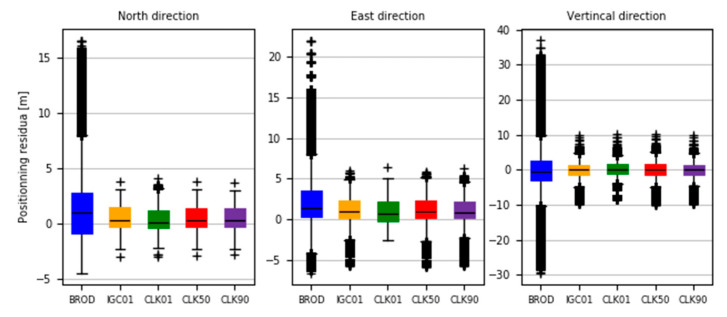
Distribution of positioning residuals in the north. east. and vertical directions.

Figure 18, Figure 19 and Figure 20 present time series of position residuals in the north. east. and vertical directions. The values obtained with the use of broadcast ephemerides are depicted in blue. while those calculated using IGC01. CLK01. CLK50. and CLK90 RTCM data streams are in orange. green. red. and violet. respectively. The use of SSR corrections results in significant improvement in position accuracy and elimination of over dozen meter outliers. The mean absolute value of horizontal position residuals for the broadcast value is 2.87 m dN and 3.07 m dE. While the same values for positions calculated using the CLK90 stream are 0.85 m and 1.34 m. respectively. The CLK90 stream shows the highest statistical accuracy; however. for the other stream. mean values of horizontal residuals are only a few centimetres higher. In the case of the vertical component. broadcast and SSR results show an even greater discrepancy. In the case of broadcast computations. mean absolute vertical residuals is 5.75 m. while in the case of the CLK90 stream. it is 1.46 m. Thus. it can be stated that the use of CLK90 SSR correction reduced the error in determining height almost four times. In the case of the other streams. mean absolute values of the vertical component are about 20 cm higher. but it is still practically 3.5 times better than that of the broadcast ephemerides. The distribution of positioning residuals is shown in Figure 21.

The analysis of the values presented in Figure 21 confirms the conclusions drawn from an analysis of previous graphs. The values of residuals obtained from positioning with the use of SSR corrections contain much fewer outliers and the median value is closer to the average value than in the case of positioning with the use of broadcast ephemerides. It is also worth noting that the standard deviation value for horizontal components was 3.59 m for broadcast positioning and 0.78 m for all SSR correction streams. For the vertical component. these values were 7.29 m and 1.34 m. respectively.

## 6. Conclusions

This paper presented attempts to evaluate the accuracy of selected SSR streams (IGC01. CLK01. CLK50. CLK90) generated by different ACs. This attempt concerned both the determination of satellite position in orbit and the correction of the satellite clock determined in real-time. On the basis of the conducted research. one can conclude that the streams calculated by specific analysis centres most often have the smallest errors relative to the final ephemeris calculated by these centres. Nevertheless. by analysing all selected streams and all reference data. it can be found that the highest accuracy of the satellite position can be obtained by using the IGC01 stream. In the case of a satellite clock correction. the differences in the considered quantities are greater. and allow to draw the first conclusions. In the case of clock errors. the CLK90 and CLK50 streams showed the highest accuracy with a slight advantage in favour of the CLK90 stream. In this case. the IGC01 stream proved to be the least accurate for all reference values. Therefore. based on the analysis of the satellite positions and the clock corrections. It is not possible to clearly identify an SSR stream that would allow obtaining of the highest accuracy.

Performing PPP allowed for better assessment of selected SSR streams. Studies carried out by the PPP method (kinematic mode) during a 33-day measurement sessions showed significant differences between the results obtained from the IGC01 stream and the other variants tested. The values obtained using IGC01 had a 2.5 times higher value of horizontal and vertical positioning residuals then in the case of the CLK90 stream. In other tested variants. the accuracy was twice better than that of IGC01. It can be concluded that care must be taken when using a stream obtained from a combined solution of other streams (IGC01) because it does not allow to obtain as high accuracy as in the case of individual results presented in the other variants (CLK01. CLK50. CLK90). The values for these variants were much better. regardless of the software used and the analysis centre. All analyses performed indicate the superiority of the CLK90 stream over the other tested variants. However. it should be noted that the advantage over the CLK50 is insignificant because the differences between these two solutions did not exceed 0.015 m.

Usually. SSR streams are considered in relation to PPP positioning and their impact on the results of computing precise position in real-time. However. in the last study. the authors decided to examine the impact of using SSR streams on the result of SPP positioning. The results of this study have shown significant improvement in determining the SPP position for all SSR streams used. The solutions applied allowed to reduce the average error value. eliminate outliers. and increase the precision of determinations by reducing the standard deviation. When determining the position. it should be mentioned that the best results were also obtained using the CLK90 stream; however. the differences range at 0.17 m. The presented results state that SSR streams do not have to be used only for advanced and technically demanding PPP positioning. but also allow for significant improvement in the quality of SPP determinations.

## Figures and Tables

**Table 1:** List of single solutions State Space Representation (SSR) corrections streams.

Stream Name	Generator	Ref Point *	Software	Stream Name	Generator	Ref Point *	Software
IGS01	ESA/ESOC	APC	RETINA	CLK30	IGS Single—Epoch	CoM	RETINA
IGC01	ESA/ESOC	CoM	RETINA	CLK31	IGS Single—Epoch	APC	RETINA
IGS02	BKG	APC	BNC	CLK35	IGS Single—Epoch	APC	BNC
IGS03	BKG	APC	BNC	CLK50	ESA/ESOC	CoM	RETINA
CLK00	BKG	CoM	RTNet	CLK51	ESA/ESOC	APC	RETINA
CLK01	BKG	CoM	RTNet	CLK52	ESA/ESOC2	CoM	RETINA
CLK10	BKG	APC	RTNet	CLK53	ESA/ESOC2	APC	RETINA
CLK11	BKG	APC	RTNet	CLK80	GMV	CoM	magicGNSS
CLK15	WHU	CoM	PANDA	CLK81	GMV	APC	magicGNSS
CLK16	WHU	APC	PANDA	CLK90	CNES	CoM	PPP—WIZARD
CLK20	DLR	APC	RETICLE	CLK91	CNES	APC	PPP—WIZARD
CLK21	DLR	CoM	RETICLE	CLK92	Phase CNES	CoM	PPP—WIZARD
CLK22	NRCan	APC	HPGNSSC	CLK93	Phase CNES	APC	PPP—WIZARD
CLK24	IGS Combination	CoM	RETINA	EUREF01	BKG	APC	BNC
CLK25	IGS Combination	APC	RETINA	EUREF02	BKG	APC	BNC

* CoM—Centre of Mass (satellite), APC – Antenna Phase Centre (satellite).

**Table 2:** Statistical summary of satellite position residuals for all selected streams.

SP3 Source	SSR Stream	Position Residuals [m]		
		Mean	Median	Standard Deviation
IGS	CLK01	0.043	0.040	0.022
IGS	CLK90	0.038	0.036	0.018
IGS	CLK50	0.033	0.030	0.016
IGS	IGC01	0.027	0.024	0.015
ESA	CLK01	0.042	0.039	0.022
ESA	CLK90	0.036	0.034	0.018
ESA	CLK50	0.032	0.032	0.018
ESA	IGC01	0.033	0.028	0.016
GFZ	CLK01	0.046	0.043	0.022
GFZ	CLK90	0.042	0.040	0.019
GFZ	CLK50	0.038	0.036	0.017
GFZ	IGC01	0.033	0.030	0.016
GRG	CLK01	0.047	0.044	0.022
GRG	CLK90	0.036	0.033	0.019
GRG	CLK50	0.040	0.044	0.017
GRG	IGC01	0.038	0.036	0.018

**Table 3:** Statistical summary of satellite clock residuals for all selected streams.

SP3 Source	SSR Stream	Clock Residuals [m]		
		Mean	Median	Standard Deviation
IGS	CLK01	0.136	0.084	0.123
IGS	CLK90	0.136	0.091	0.106
IGS	CLK50	0.106	0.064	0.130
IGS	IGC01	0.174	0.205	0.124
ESA	CLK01	0.040	0.034	0.028
ESA	CLK90	0.040	0.034	0.027
ESA	CLK50	0.036	0.031	0.029
ESA	IGC01	0.047	0.046	0.029
GFZ	CLK01	0.024	0.018	0.023
GFZ	CLK90	0.020	0.017	0.018
GFZ	CLK50	0.023	0.018	0.022
GFZ	IGC01	0.029	0.024	0.022
GRG	CLK01	0.023	0.017	0.028
GRG	CLK90	0.018	0.015	0.016
GRG	CLK50	0.023	0.018	0.028
GRG	IGC01	0.028	0.021	0.021

**Table 4:** PPP processing parameters used in the study.

Parameter	Value
GNSS system and signals	GPS P3 code iono-free linear combination GPS L3 phase iono-free linear combination
Troposphere model ANTEX file	UNB3m IGS14
Satellite orbits and clocks	1. IGC01 2. CLK01 3. CLK50 4. CLK90
Code Sigma [m]	2.0
Phase Sigma [m]	0.005
Max Code Residual [m]	4.0
Max Phase Residual [m]	0.02
Data interval [s]	1
Session length [s]	2 851 200
Min # of satellites	5
Min satellite elevation [deg]	5
Min satellite SNR	40

**Table 5:** Positioning RMS [m] of the PPP results.

Stream	IGC01				CLK01				CLK50				CLK90			
RMS [m]	dN	dE	dV	3D	dN	dE	dV	3D	dN	dE	dV	3D	dN	dE	dV	3D
HERT	0.13	0.16	0.23	**0.31**	0.02	0.04	0.05	**0.07**	0.04	0.05	0.06	**0.09**	0.02	0.04	0.05	**0.07**
YELL	0.16	0.15	0.23	**0.32**	0.04	0.04	0.07	**0.09**	0.03	0.03	0.04	**0.06**	0.03	0.03	0.03	**0.05**
BRAZ	0.12	0.21	0.32	**0.40**	0.06	0.12	0.17	**0.22**	0.03	0.05	0.11	**0.12**	0.03	0.06	0.13	**0.15**
OHI3	0.11	0.13	0.22	**0.28**	0.08	0.09	0.16	**0.20**	0.03	0.03	0.13	**0.14**	0.03	0.04	0.13	**0.14**
KERG	0.11	0.14	0.25	**0.31**	0.06	0.10	0.14	**0.18**	0.04	0.05	0.10	**0.12**	0.04	0.06	0.10	**0.12**
MAR6	0.09	0.10	0.15	**0.20**	0.03	0.04	0.05	**0.07**	0.04	0.04	0.05	**0.07**	0.03	0.03	0.05	**0.07**
JOZ2	0.09	0.14	0.16	**0.24**	0.03	0.05	0.05	**0.08**	0.03	0.06	0.05	**0.09**	0.03	0.04	0.05	**0.07**
URUM	0.19	0.36	0.45	**0.61**	0.17	0.31	0.35	**0.50**	0.14	0.29	0.32	**0.45**	0.10	0.24	0.24	**0.35**
MAC1	0.11	0.13	0.19	**0.26**	0.03	0.06	0.08	**0.10**	0.03	0.04	0.07	**0.08**	0.03	0.04	0.06	**0.07**
TOW2	0.11	0.15	0.24	**0.30**	0.09	0.08	0.18	**0.22**	0.11	0.10	0.19	**0.24**	0.09	0.08	0.17	**0.21**
Mean	**0.12**	**0.16**	**0.24**	**0.32**	**0.06**	**0.09**	**0.13**	**0.17**	**0.05**	**0.07**	**0.11**	**0.15**	**0.04**	**0.07**	**0.10**	**0.13**
